# Electrodeposition of Polypyrrole and Reduced Graphene Oxide onto Carbon Bundle Fibre as Electrode for Supercapacitor

**DOI:** 10.1186/s11671-017-2010-3

**Published:** 2017-04-04

**Authors:** Hamra Assyaima Abdul Bashid, Hong Ngee Lim, Sazlinda Kamaruzaman, Suraya Abdul Rashid, Robiah Yunus, Nay Ming Huang, Chun Yang Yin, Mohammad Mahbubur Rahman, Mohammednoor Altarawneh, Zhong Tao Jiang, Pandikumar Alagarsamy

**Affiliations:** 1 Department of Chemistry, Faculty of Science, Universiti Putra Malaysia, 43400 UPM Serdang, Selangor Malaysia; 2 Functional Device Laboratory, Institute of Advanced Technology, Universiti Putra Malaysia, 43400 UPM Serdang, Selangor Malaysia; 3 Department of Chemical and Environmental Engineering, Faculty of Engineering, Universiti Putra Malaysia, 43400 UPM Serdang, Selangor Malaysia; 4 Materials Processing and Technology Laboratory (Nanomaterials and Nanotechnology Group), Institute of Advanced Technology, Universiti Putra Malaysia, 43400 UPM Serdang, Selangor Malaysia; 5 Faculty of Engineering, Xiamen University of Malaysia, Jalan Sunsuria, Bandar Sunsuria, 43900 Sepang, Selangor Darul Ehsan Malaysia; 6 Newcastle University Singapore, SIT Building @ Ngee Ann Polytechnic, 537 Clementi Road #06-01, Singapore, 599493 Singapore; 7 Department of Physics, Jahangirnagar University, Savar, Dhaka, 1342 Bangladesh; 8 Surface Analysis and Materials Engineering Research Group, School of Engineering and Information Technology, Murdoch University, Murdoch, WA 6150 Australia; 9 Research Institute & Department of Chemistry, SRM University, Kattankulathur-603 203, Chennai, India

**Keywords:** Carbon bundle fibre, Graphene, Polypyrrole, Flexible supercapacitor

## Abstract

**Abstract:**

A nanocomposite comprising of polypyrrole and reduced graphene oxide was electrodeposited onto a carbon bundle fibre (CBF) through a two-step approach (CBF/PPy-rGO-2). The CBF/PPy-rGO-2 had a highly porous structure compared to a nanocomposite of polypyrrole and reduced graphene oxide that was electrodeposited onto a CBF in a one-step approach (CBF/PPy-rGO), as observed through a field emission scanning electron microscope. An X-ray photoelectron spectroscopic analysis revealed the presence of hydrogen bond between the oxide functional groups of rGO and the amine groups of PPy in PPy-rGO-2 nanocomposite. The fabricated CBF/PPy-rGO-2 nanocomposite material was used as an electrode material in a symmetrical solid-state supercapacitor, and the device yielded a specific capacitance, energy density and power density of 96.16 F g^− 1^, 13.35 Wh kg^− 1^ and of 322.85 W kg^− 1^, respectively. Moreover, the CBF/PPy-rGO-2 showed the capacitance retention of 71% after 500 consecutive charge/discharge cycles at a current density of 1 A g^− 1^. The existence of a high degree of porosity in CBF/PPy-rGO-2 significantly improved the conductivity and facilitated the ionic penetration. The CBF/PPy-rGO-2-based symmetrical solid-state supercapacitor device demonstrated outstanding pliability because the cyclic voltammetric curves remained the same upon bending at various angles.

**Graphical Abstract:**

Carbon bundle fibre modified with porous polypyrrole/reduced graphene oxide nanocomposite for flexible miniature solid-state supercapacitor.

**Electronic supplementary material:**

The online version of this article (doi:10.1186/s11671-017-2010-3) contains supplementary material, which is available to authorized users.

## Background

The rapid growth of next-generation portable electronics has led to intensive efforts to develop supercapacitors with flexible, rigid, small, lightweight, eco-friendly and high storage capacity [1]. Supercapacitors, which are also known as electrochemical capacitors, offer a promising alternative approach to energy storage devices because of their ability to store and deliver a high power density, and long life cycle with short charging time, simply by utilising the charge separation of the electrochemical interface between the electrode and electrolyte [2–4]. The conventional two-electrode system supercapacitors are planar-structured, consisting of two active electrodes kept apart by an electrolyte as an indispensable and electrically insulating separator [5]. The supercapacitors are large, bulky and heavy, and are not suitable for portable electronic devices. Thus, to address this issue, much effort has been devoted to the development of fibre- or wire-shaped supercapacitors that are flexible, lightweight and easily shaped in portable electronic devices [6–8].

Fibre- or wire-shaped supercapacitors are commonly built on fibrous or interwoven substrates and can be directly integrated into a wearable and embedded device units in sensors, environmental monitoring, display and implanted medical devices [9]. Metal-based fibres such as aluminium wires have previously been used as a current collector or core electrode because of its high conductivity and ease of availability. However, the performance is limited due to its heaviness and is easily oxidised under ambient conditions [6, 10]. Carbon-based fibres, like carbon microfibres and graphene fibres, have been used to replace metal-based fibres owing to its great flexibility, light weight, high mechanical strength, high conductivity and stability under ambient conditions [6, 11].

The choice of electro-active materials also plays important roles in determining the electrochemical performances of supercapacitor devices. Graphene has been studied extensively as an electro-active material for supercapacitors due to its promising properties such as large (theoretical) surface areas, high charge carrier mobility, excellent conductivity, high mechanical strength, and extremely high thermal conductivity, with the ability to store and release energy through the separation of electronic and ionic charges in the electrode and electrolyte interface [12–15]. In particular, reduced graphene oxide (rGO) is often used instead of graphene, mainly because it can be ubiquitously produced from graphene oxide (GO) through various methods such as hydrothermal reaction, laser irradiation and chemical or electrochemical reduction under mild conditions [16]. Moreover, using GO as a starting material can provide good dispersion stability and prevent aggregation in the reaction solution [17].

Simultaneously, electrically conducting polymers such as polypyrrole (PPy) have been studied extensively as pseudocapacitor materials for supercapacitors since they offer good electrical conductivity, high charge densities, low cost and excellent pseudocapacitor behaviours [18–20]. Furthermore, the PPy also provides a greater degree of flexibility in electrochemical processing [21, 22]. Conducting polymers can improve the device by undergoing a redox reaction to store a charge in the bulk of the material and hence increase the energy stored and reduce self-discharge [23, 24]. Recently, the hybridization of carbon-based materials and conducting polymers is believed to be able to enhance the capacitance and stability of a supercapacitor performance through the favourable synergistic effect between them [25, 26].

This study focused on fabrication of flexible symmetrical solid-state supercapacitors in which two carbon bundle fibre (CBF) electrodes were assembled into a supercapacitor device by using them to sandwich polyvinyl alcohol-potassium acetate (PVA-CH_3_CO_2_K), which served as an indispensable solid-state electrolyte. The CBF served as a flexible current collector with electro-active materials, while the rGO and PPy were electrochemically deposited on it at a constant potential. The presence of the catalyst in the aqueous solution (PPy-rGO-2) during the electrodeposition was compared to those of PPy and PPy-rGO to investigate the influences of the catalyst on the surface morphology and electrochemical capacitive performance. These symmetrical solid-state supercapacitors inherited flexibility while maintaining high capacitive performances.

## Methods

Graphite powder was purchased from Asbury Graphite Mills Inc. (code no. 3061). Sulfuric acid (H_2_SO_4_), phosphoric acid (H_3_PO_4_), potassium permanganate (KMnO_4_) and hydrogen peroxide (H_2_O_2_) were purchased from Systerm Chemicals, Malaysia. Hydrochloric acid (HCl) and iron (III) chloride (FeCl_3_) were purchased from Sigma-Aldrich, while potassium acetate (CH_3_CO_2_K) was purchased from BDH Reagents and Chemicals. Sodium *p*-toluenesulfonate (NapTs), poly(vinyl alcohol) (PVA) flakes (MW = 60,000) and glycerol purchased from Merck, followed by pyrrole (99%) purchased from Acros Organic, were stored at 0 °C and distilled before use. Hydrophilic carbon cloth (ELAT) was purchased from NuVant Systems Inc., USA.

**Scheme 1:**
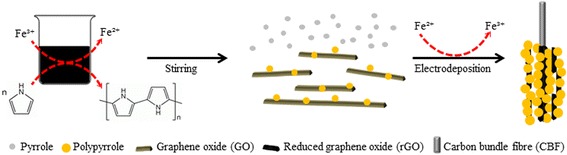
Schematic diagram of the synthesis process of CBF/PPy-rGO-2

The CBF/PPy-rGO-2 nanocomposite modified electrode was fabricated by a two-step approach process (Scheme 1). In a typical fabrication method, initially, graphene oxide (GO) was prepared through the modified Hummer’s method [27]. Then, a PPy-rGO-2 nanocomposite was electrochemically deposited on a CBF using a one-compartment cell with potentiostat/galvanostat (Princeton Applied Research), following our previous work with slight modification [28]. The aqueous solution contained a mixture of 1.0 mM FeCl_3_, 1.0 mg/mL GO, 0.1 M pyrrole and 0.1 M NapTS, and was vigorously stirred for 5 min. A CBF was used as a working electrode, and platinum (Pt) rod and saturated calomel electrode (SCE) were used as counter and reference electrodes, respectively. The electrochemical deposition was carried out at a constant potential of +0.8 V (versus SCE) at room temperature for 5 min. For comparison, a PPy-rGO nanocomposite and PPy nanocomposite without the catalyst were synthesised. All of the as-prepared electrodes were washed with distilled water and dried under ambient conditions before proceeding to the fabrication of the symmetrical solid-state supercapacitor device.

The solid-state electrolyte was prepared using 10% (*w*/*v*) PVA in water and stirred continuously at 100 °C until complete solvation. The potassium acetate (1.96 g) was added to the solution and mixed thoroughly. This was followed by glycerine (10% *w*/*w*) which was added as a plasticiser to prevent the loss of electrolyte. Then, the as-prepared CBF/PPy-rGO-2 was used as an electrode for the fabrication of a symmetrical solid-state supercapacitor device. The two symmetrical electrodes were dipped in the solid-state electrolyte and sandwiched together side by side which served as positive and negative electrodes. Meanwhile, the solid-state electrolyte acted as both the electrolyte and ion porous separator, and the fabricated device was left to dry at room temperature.

The cross sections and surface morphologies of the as-prepared electrodes were studied using an FEI Quanta 400F field emission scanning electron microscope (FESEM). The core-level valence electrons of the sample and the atomic percentages of the existing elements were characterised by X-ray photoelectron spectroscopy (XPS), using a Kratos Axis Ultra XPS spectrometer (Manchester, UK) with an Al-K_α_ monochromatic radiation source (hν = 1486.6 eV, 10 mA emission current, and 15 kV accelerating voltage). The electrochemical performances of the as-fabricated CBF/PPy-rGO-2 electrode-based symmetrical solid-state supercapacitor devices were investigated using the potentiostat/galvanostat (Princeton Applied Research). Electrochemical impedance spectroscopy (EIS) measurements were performed between 5 mHz and 100 kHz, with an AC amplitude of 5 mV. Cyclic voltammetry (CV) and galvanostatic charge/discharge (GCD) analyses were performed at a working potential of 0–1 V. The specific capacitance in farads per gram (Cm) of the supercapacitor device was calculated using integration on the area under a CV curve (Eq. 1) and the slope of the discharge curve of the GCD (Eq. 2) [29].

1 $$ C m = k\frac{{\displaystyle \int } i}{ms} $$

2 $$ C m = k\frac{it}{\varDelta v. m} $$

where ∫*i* is the integrated area of the CV curve, *m* is the mass of the electro-active materials in grams, *s* is the scan rate of the CV conducted, *i* is the applied current, *t* is the elapsed time during the discharged process, ∆*v* is the total working potential and *k* is a constant multiplier (*k* = 2 if the mass of a single electrode is used and *k* = 4 if the mass of both electrodes is taken into account). The energy densities and power densities of the supercapacitor devices were obtained from Eqs. 3 and 4, respectively [30].

3 $$ {E}_{\mathrm{cell}} = \frac{1}{2}{C}_{\mathrm{cell}}{V}^2 $$

4 $$ {P}_{\mathrm{cell}} = \frac{E_{\mathrm{cell}}}{\varDelta t} $$

where *C*_cell_ is the specific capacitance of the cell from a charge/discharge calculation (*F g* ^− 1^), *V* is the potential window (*V*) and ∆*t* is the discharge time (s).

## Results and Discussion

**Fig. 1:**
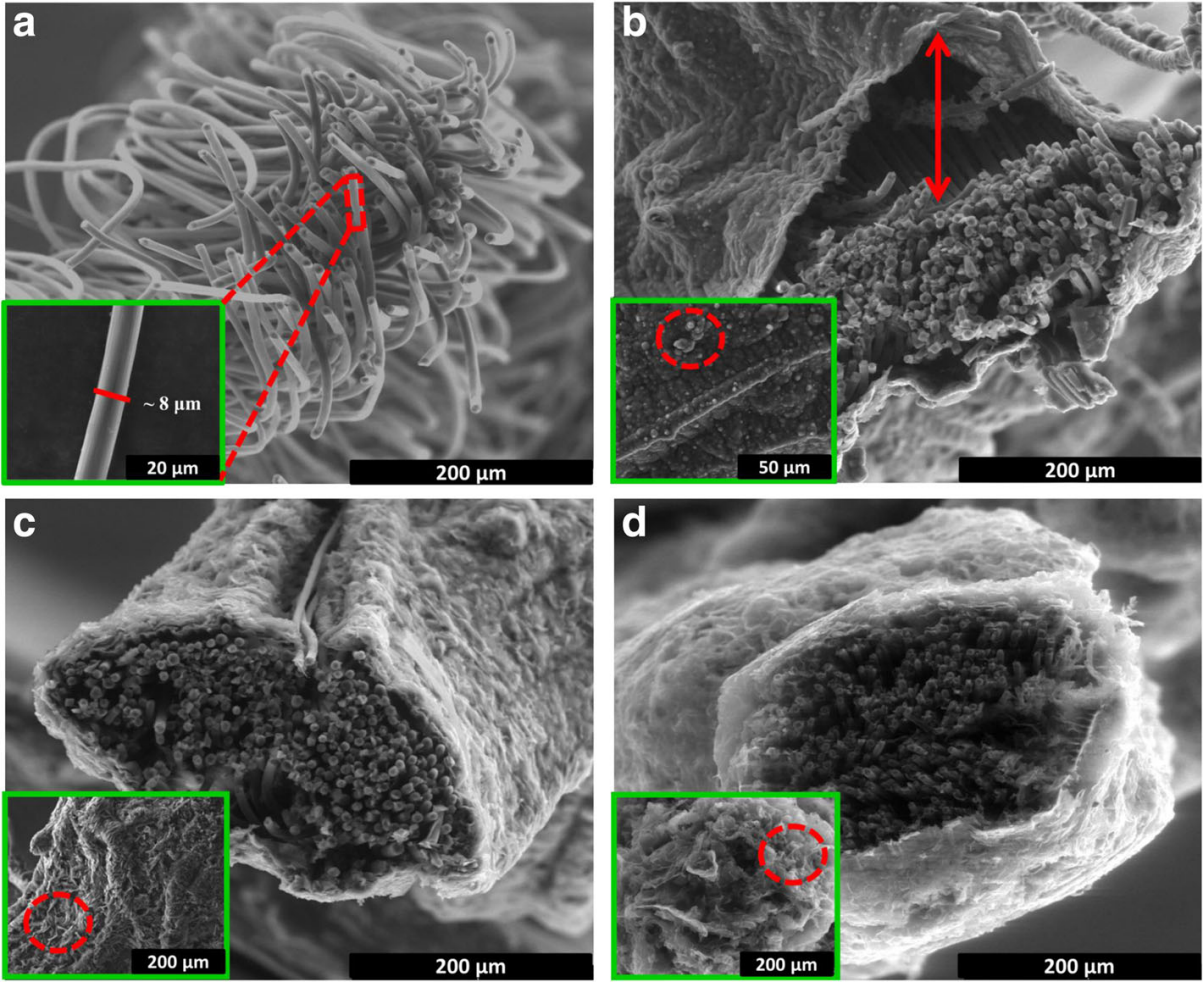
Cross-sectional FESEM images along with images of surface morphologies (*inset*) of as-prepared electrodes. **a** Bare CBF. **b** CBF/PPy. **c** CBF/PPy-rGO. **d** CBF/PPy-rGO-2

In Fig. 1a, the bare CBF consists of a bundle of carbon fibre with a clear and smooth surface, and for a single carbon fibre has a diameter of ~8 μm. During the electrochemical deposition, the electro-active materials were only deposited on the outer surface of CBF, which can be seen from the FESEM cross-sectional images. The PPy has a typical bulbous and cauliflower morphology, as depicted in Fig. 1b, while Fig. 1c shows that the PPy-rGO generated an almost flat surface with some creases easily found [17]. The morphology of the PPy changed drastically when GO was introduced into the aqueous solution, with no clear distinction between the PPy and GO [31]. In the presence of the catalyst, the PPy-rGO-2 images reveal a highly porous network with an extended and continuous morphology [32], as displayed in Fig. 1d.

The difference in the surface morphologies of the PPy-rGO and PPy-rGO-2 compared to that with PPy alone can be explained by the formation mechanism for the PPy on the rGO sheets during the potentiostatic electropolymerisation and catalyst-assisted electrodeposition process. The one-step approach of the potentiostatic electropolymerisation process for PPy-rGO resulted in a continuous layer-by-layer deposition of PPy and GO, where negatively charged GO would be attracted to the pyrrole radical cations during the electrodeposition, and a new layer of PPy would form when the existing GO was fully occupied by PPy [33]. The reaction consequently removed oxygen from GO, thus converting GO into rGO [31]. In contrast, the catalyst-assisted electrodeposition process for PPy-rGO-2 involved a two-step approach, in which during the stirring process, the catalyst oxidised the pyrrole monomers initially to form PPy nanoparticles on the GO sheets (PPy-GO). The subsequent electrodeposition process increased the size and amount of PPy and then bound the sheets to one another and reduced the GO to rGO, which resulted in a maximum exposed area of PPy and prevented the rGO from restacking with the neighbouring rGO [28]. The highly porous structure of PPy-rGO-2 facilitated the electrolyte penetration and eventually increased the specific capacitance value.

**Fig. 2:**
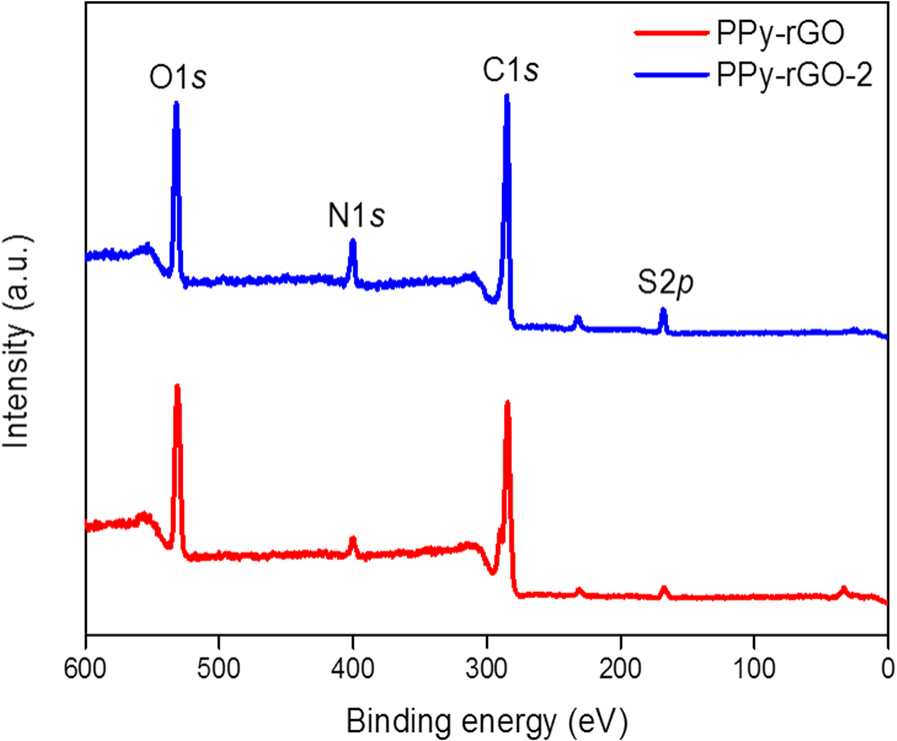
XPS survey scans of PPy-rGO and PPy-rGO-2 nanocomposites

Figure 2 shows the XPS survey spectra of the PPy-rGO and PPy-rGO-2 nanocomposites. It illustrates the presence of oxygen, nitrogen, carbon and sulphur in the PPy-rGO and PPy-rGO-2 nanocomposites. The details of the atomic compositions of the PPy-rGO and PPy-rGO-2 nanocomposites are listed in Table 1. The carbon and nitrogen elements arose from the PPy backbone, whereas the sulphur came from the NapTS used as a dopant in the polymerisation process. Of course, a major portion of the carbon also came from the graphene oxide. However, the oxygen most likely originated from the surface oxidation of the PPy. These results were found to be consistent with the previous report on electrochemically deposited PPy nanoparticle incorporated rGO [34].

**Table 1:** Elemental compositions of fabricated nanocomposites

Nanocomposite	Element	Binding energy (eV)	Atomic percentages (at.%)
PPy-rGO	S	168.5	1.98
	C	284.5	75.08
	N	400.0	2.58
	O	532.0	20.35
PPy-rGO-2	S	167.5	3.53
	C	285.0	71.00
	N	399.5	6.79
	O	531.5	18.69

**Fig. 3:**
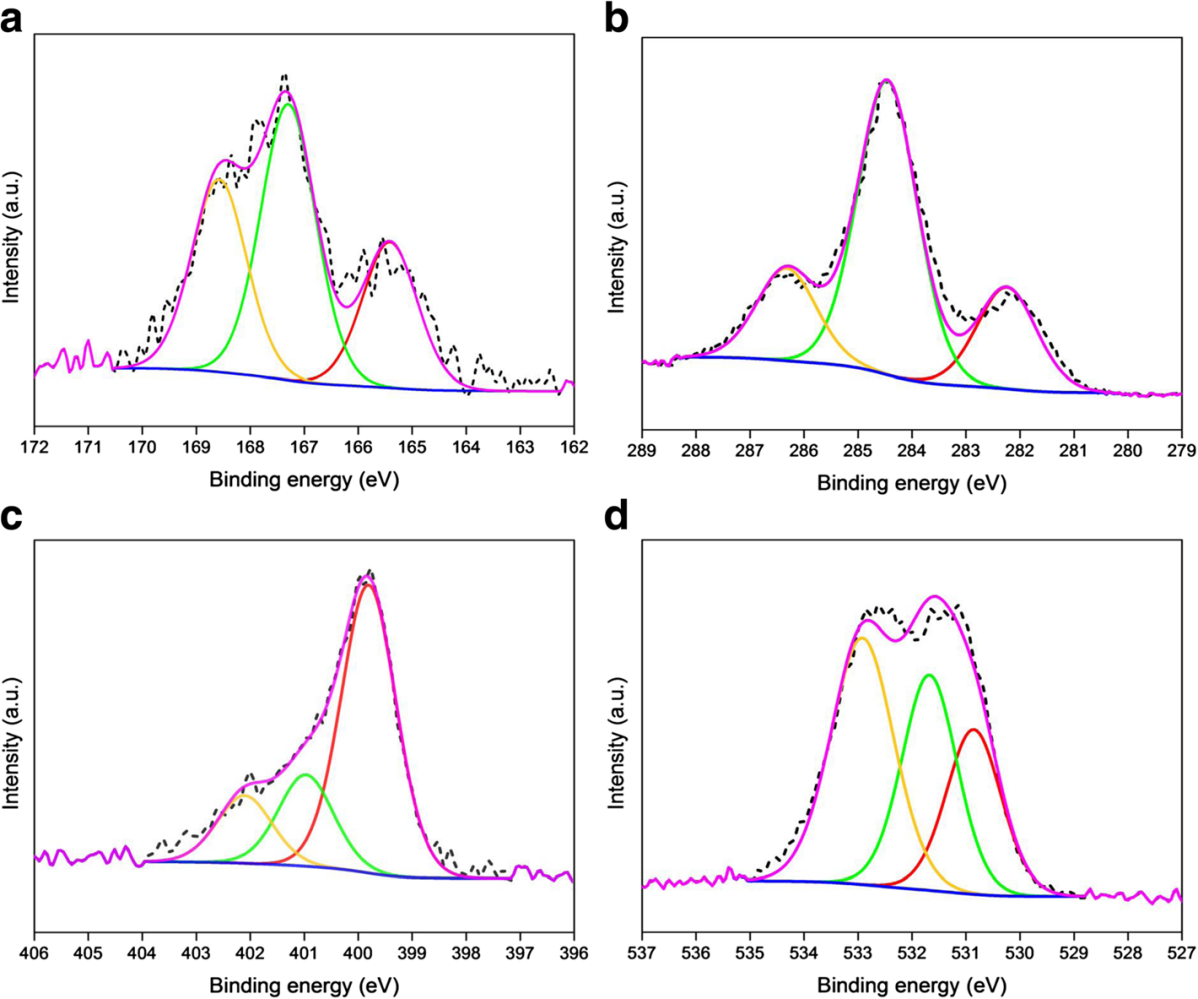
XPS core-level spectra of PPy-rGO nanocomposite and corresponding deconvolutions of **a** S2p, **b** C1s, **c** N1s and **d** O1s states. *Dotted lines* show raw data, and *solid lines* are fitting curves

**Fig. 4:**
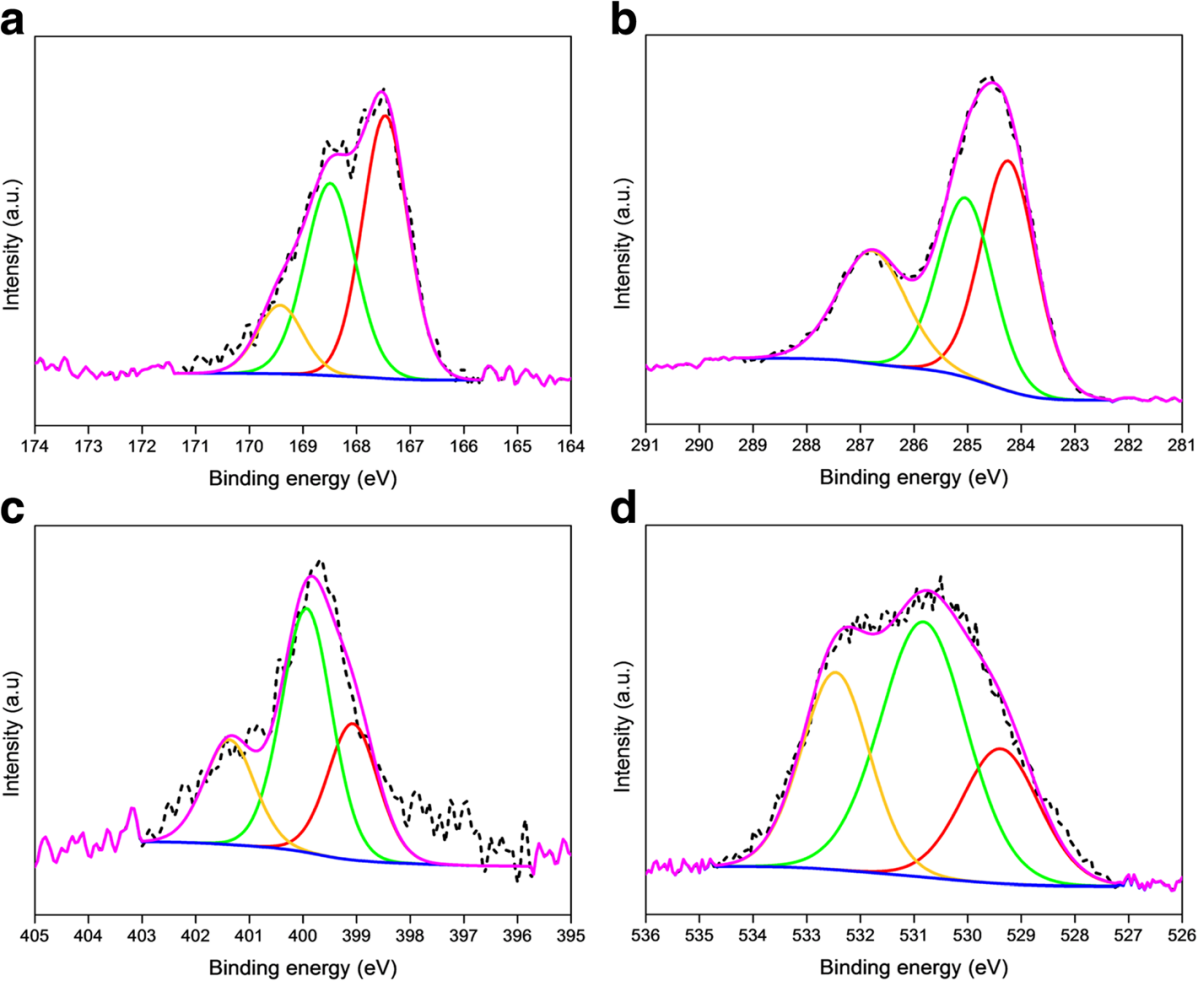
XPS core-level spectra of PPy-rGO-2 nanocomposite and corresponding deconvolutions of **a** S2p, **b** C1s, **c** N1s and **d** O1s states. *Dotted lines* show raw data, and *solid lines* are fitting curves

High-resolution XPS measurements were performed to comprehend the possible chemical bonding, including oxidation and states of the PPy-rGO and PPy-rGO-2 nanocomposites, as well as to estimate their compositions. Figures 3 and 4 show the deconvolution of high-resolution S2p, C1s, N1s and O1s core-level spectra and the relative binding energies for PPy-rGO and PPy-rGO-2, respectively. The details of the chemical bonding states, photoelectron line positions and percentages of the deconvoluted components from the high-resolution S2p, C1s, N1s and O1s spectra are presented in Table 2.

**Table 2:** Curve fitting results for core-level binding energies of fabricated nanocomposites

	Core-level	Bonding states	Binding energy (eV)	FWHM (eV)	Percentages of the component (%)
PPy-rGO	S2p	C_4_H_4_S	165.4	1.2	23.75
		S–O–	166.9	1.2	44.88
		SO_2_	168.5	1.2	31.36
	C1s	Carboxyl group	282.4	1.3	20.81
		Steady peak position π-π interactions	284.6	1.3	60.24
		C–S/C=O/C=N/=C–NH^+^ bonds	286.4	1.3	18.95
	N1s	–NH–	399.7	1.2	64.39
		=C–NH^+^– (polaron)	400.9	1.2	20.46
		=NH^+^– (bipolaron)	402.1	1.2	15.15
	O1s	C=O/S=O/O=C/HO–C bonds	530.8	1.2	24.87
		C=O/O–C=O bonds	531.6	1.2	32.52
		O–C/C–O–C/COOH/C–OH/H_2_O bonds	532.9	1.4	42.61
PPy-rGO-2	S2p	C_4_H_4_S	167.5	1.0	48.43
		S–O–	168.5	1.1	38.92
		SO_2_	169.4	1.0	12.56
	C1s	Carboxyl group	284.2	1.2	41.81
		Steady peak position π-π interactions	285.1	1.2	32.58
		C–S/C=O/C=N/=C–NH^+^ bonds	286.8	1.4	25.60
	N1s	–NH–	399.2	1.1	28.14
		=C–NH^+^– (polaron)	400.0	1.1	50.24
		=NH^+^– (bipolaron)	401.4	1.1	21.62
	O1s	C=O/S=O/O=C/HO–C bonds	529.5	1.6	22.74
		C=O/O–C=O bonds	530.9	1.9	47.96
		O–C/C–O–C/COOH/C–OH/H_2_O bonds	532.5	1.5	29.30

Figure 3a demonstrates the deconvolutions of the high-resolution XPS spectrum of the S2p photoelectron line for PPy-rGO. The S2p can be resolved into three components at 165.4, 166.9 and 168.5 eV due to the contributions from the C_4_H_4_S, S–O– and SO_2_ species, respectively. This shows that the occurrence of these salient chemical bonding states is in good agreement with the NIST database (NIST Standard Reference Database 20, Version 4.1). The curve fitting of the high-resolution C1s photoelectron line is depicted in Fig. 3b. The deconvoluted C1s spectrum is subdivided into three fragments at three different binding energies of 282.4, 284.6 and 286.4 eV. The first two components are attributed to the presence of the carboxyl group bonding state and steady peak position π-π interactions in pyrrole rings, respectively. The third component at 286.4 eV suggests the possible occurrence of C–S/C=O/C=N/=C–NH^+^ bonding structures in the nanocomposites. The deconvolution of the N1s peak in the XPS spectrum offers three components with remarkably different intensities, as shown in Fig. 3c. The first peak detected at 399.7 eV is ascribed to neutral nitrogen in the pyrrole ring (–NH). The second component detected at 400.9 eV corresponds to the polaron state (–NH^+^–), and another peak at a relatively higher binding energy (402.1 eV) can be assigned to =NH^+^–, which may have originated from the presence of bipolaron charge carriers [35]. Figure 3d shows the deconvolution of the O1s spectrum, where three segments are located at binding energy positions of 530.8, 531.6 and 532.9 eV, suggesting the presence of C=O/S=O/O=C/HO–C bonds, C=O/O–C=O bonds and O–C/C–O–C/COOH/C–OH/H_2_O bonds, respectively. The C–O and COOH bonds are offcuts of the oxide functional groups of graphene oxide, whereas the S–O bond originates from the NapTS used in the polymerisation process.

The as-stated three component peaks of the S2p photoelectron line for PPy-rGO-2 could be fitted to the binding energies of 167.5 eV (C_4_H_4_S), 168.5 eV (S–O–) and 169.4 eV (SO_2_), respectively, as depicted in Fig. 4a. The high-resolution XPS spectra of C1s and N1s are imperative because the shift in the photoelectron line position indicates a difference in the electron density of the neighbouring atoms via the existing chemical bonding states [36]. In the PPy-rGO, the principal C1s peak at a binding energy of 284.6 eV is related to the steady peak position π-π interactions, which is consistent with an earlier report [37]. However, for the PPy-rGO-2, the main C1s peak was found to shift slightly (0.5 eV) towards a higher binding energy, as depicted in Fig. 4b. The shift indicates a possible electronic disorder produced via new chemical bonds, which refer to the hydrogen-bridge bond between the oxygen containing the functional group of carbon and the NH-group of PPy [38]. For this hydrogen-bridge bond, electrons transfer from the C to O atoms of the carbon functional groups and the NH-group of the PPy. As a result, the reduced electron density of the C atoms confirms a progressive transfer of the C1s XPS spectrum. However, for the N1s spectrum, the reverse phenomenon is expected because in N atoms, the electron density rises over the creation of the hydrogen bonds. This feature is clearly seen in Fig. 4c and Table 2. This negative shift of the N1s spectrum indicates the electron donation through the oxygen functionalities of carbon, which is responsible for the electron dislocations in the vicinity of the nitrogen atoms [39, 40]. A similar feature is also noticed for the O1s spectrum in Fig. 4d for the PPy-rGO-2 sample.

**Fig. 5:**
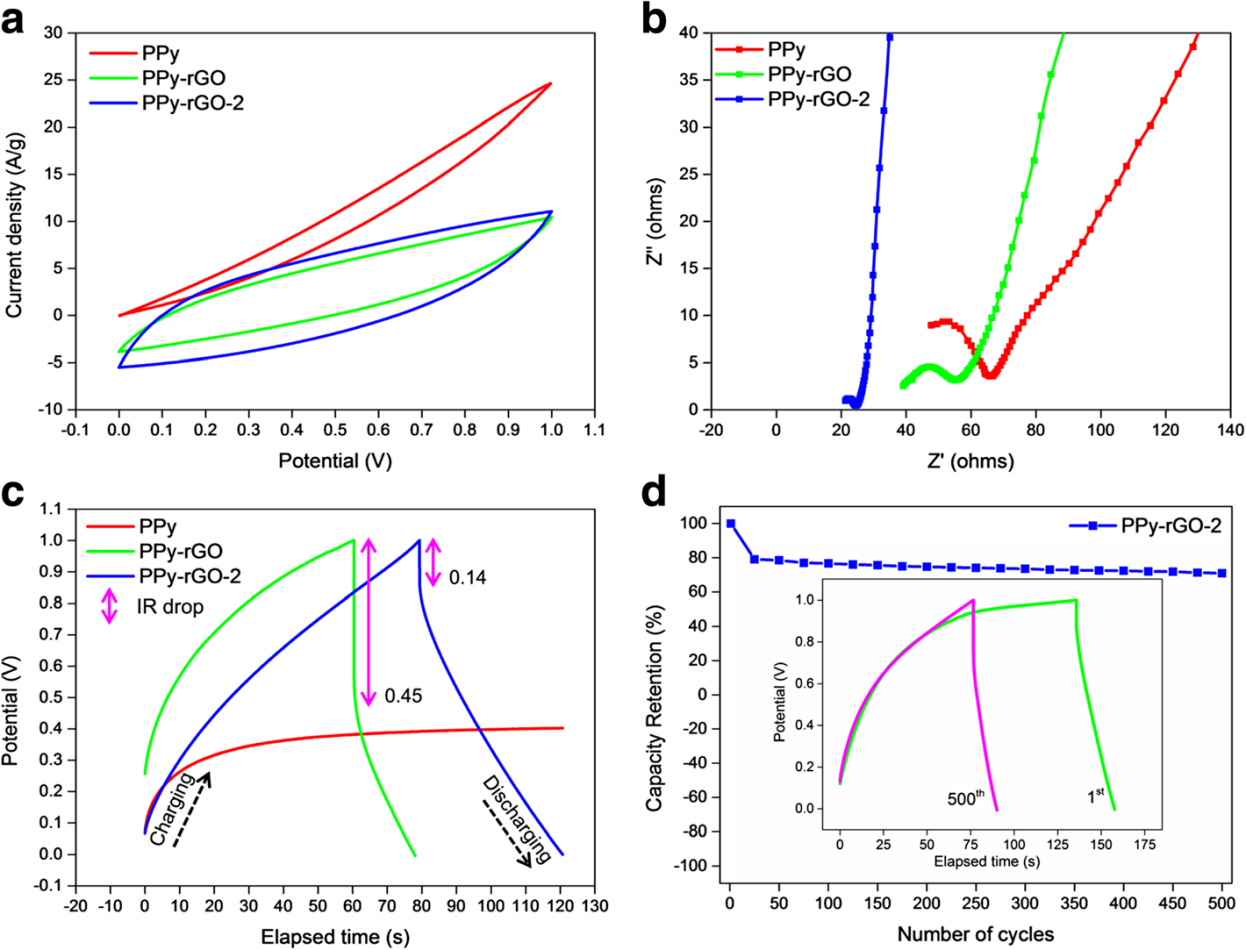
Electrochemical performances of CBF/PPy, CBF/PPy-rGO and CBF/PPy-rGO-2 modified carbon bundle fibre-based symmetrical solid-state supercapacitor devices. **a** CV curves at scan rate of 100 mV s^− 1^. **b** Nyquist plots. **c** Galvanostatic charge/discharge profiles at a current density of 1 A g^− 1^. **d** Capacity retention of CBF/PPy-rGO-2 supercapacitor device at a current density of 1 A g^− 1^, with their respective curves shown in the *inset*

Cyclic voltammetry (CV) measurements were carried out to evaluate the electrochemical performance of the supercapacitor devices. The CBF/PPy, CBF/PPy-rGO and CBF/PPy-rGO-2 supercapacitor devices had pseudo-rectangular-shaped cyclic voltammogram without any observable redox peaks at a scan rate of 100 mV s^− 1^ as shown in Fig. 5a, indicating a good capacitive behaviour for the supercapacitors while using the CBF as the current collector [41]. The specific capacitance of the CBF/PPy-rGO-2 was 130.81 F g^− 1^, which was 1.56 and 3.37 times higher than that of CBF/PPy-rGO (83.58 F g^− 1^) and CBF/PPy (38.78 F g^− 1^), respectively, due to the improved conductivity and diffusion of the ions into the electro-active materials present on the electrode surface. It can be proven by the equivalent series resistance (ESR) obtained from the first *x*-intercept value and the slope (tan *θ*) of the Nyquist plot [6] in Fig. 5b, based on EIS measurements. The ESR values of the CBF/PPy, CBF/PPy-rGO and CBF/PPy-rGO-2 were 47.75, 39.09 and 21.30 Ω, respectively, revealing that the presence of the rGO improved the conductivity of the PPy. The calculated Nyquist plot slope value is theoretically parallel to the imaginary axis (vertical line), indicating a real capacitive behaviour and low ionic diffusion resistance within the electrode structure [41]. The Nyquist plot of CBF/PPy-rGO-2 is almost a straight line, ~90°, as compared to that of CBF/PPy and CBF/PPy-rGO, resulting in a good diffusion of the electrolyte ions in the PPy-rGO-2 electrodes.

Moreover, the charge transfer resistance (R_ct_) can also be calculated from the diameter of the semicircle formed by the Nyquist plot, which relates to the interfacial processes of the counter-ions through the electrode/electrolyte interface [31]. The R_ct_ values of the PPy, PPy-rGO and PPy-rGO-2 modified electrodes were 33.38, 22.57 and 4.85 Ω, respectively, revealing that among the investigated electrodes, the PPy-rGO-2 modified electrode had the lowest interfacial resistance with a good charge propagation behaviour. Moreover, CBF/PPy-rGO-2 had a highly porous structure, which enabled easier access for the electrolyte ions, resulting in less resistance in the electrode [28]. Thus, the high conductivity and good diffusion of the electrolyte contributed to a higher specific capacitance value.

Galvanostatic charge/discharge (GCD) test for the supercapacitor devices was performed at a constant current density of 1 A g^− 1^. The CBF/PPy supercapacitor device failed to charge up to the highest applied potential (1.0 V), as depicted in Fig. 5c. This phenomenon could be attributed to the gap between the deposited layer and the current collector, as shown in Fig. 1b, which eventually disrupted the penetration of the electrolyte and the faradaic charging/discharging between the electrolyte and electrode. In contrast, the CBF/PPy-rGO and CBF/PPy-rGO-2 had asymmetrical charge and discharge curves, which imply pseudo-capacitance behaviours [42]. In addition, the IR drops were due to the presence of internal resistance in the electrode associated with the electrical connection resistance, bulk solution resistance and resistance of ion migration in the electrode, which contributed to the non-linear discharge curves [43]. As seen in Fig. 5c, CBF/PPy-rGO-2 had a lower IR drop than CBF/PPy-rGO at the beginning of the discharge process, indicating better charge efficiency [44]. The specific capacitance of the CBF/PPy-rGO-2 supercapacitor device was 96.16 F g^− 1^, which was 1.51-fold higher than that of CBF/PPy-rGO (63.27 F g^− 1^). The CBF/PPy-rGO-2 supercapacitor device had an energy density and power density of 13.35 Wh kg^− 1^ and 322.85 W kg^− 1^, respectively.

In order to evaluate the cycling stability of the CBF/PPy-rGO-2 supercapacitor device, GCD studies were performed at a relatively high current density of 1 A g^− 1^, and the capacity retention as a function of the number of GCD cycles is presented in Fig. 5d. During the first 25 cycles of GCD, the capacity retention decreased by about 21%, presumably related to the low electrochemical stability of PPy, degradation of the polymer chain and deterioration of the electro-active materials after an abrupt and excessive swelling and shrinking process during the GCD cycles [27, 45]. Encouragingly, the GCD cycles stabilised thereafter, and after 500 cycles, the CBF/PPy-rGO-2 had a capacity retention of 71% at a current density of 1 A g^− 1^.

**Fig. 6:**
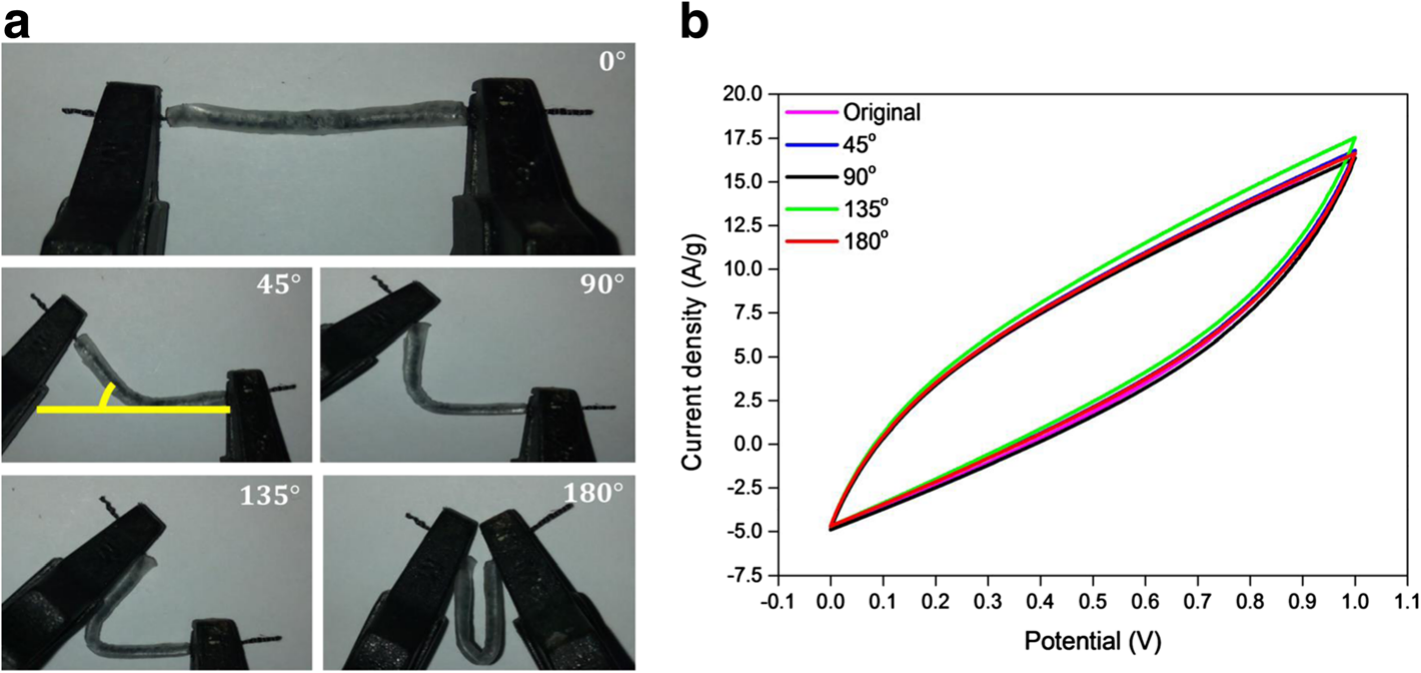
**a** Images of flexible supercapacitor device and **b** CV curves of CBF/PPy-rGO-2 supercapacitor devices bent at various angles at a scan rate of 100 mV s^− 1^

To investigate the mechanical bendability/flexibility of the as-fabricated supercapacitor device and its effect on the capacitance, the CBF/PPy-rGO-2 was bent at various angles, as depicted in Fig. 6. Interestingly, shape and size of the CV curves were almost the same at all angles at a scan rate of 100 mV s^− 1^, revealing that the bending had nearly no effect on the specific capacitance values. The combination of solid-state electrolyte and flexible current collector with flexible free-standing electro-active materials made the CBF/PPy-rGO-2 supercapacitor device capable of withstanding stress with no drastic changes in its electrochemical performance, demonstrating an excellent mechanical bendability. The good cycle stability and excellent flexibility of the CBF/PPy-rGO-2 also further demonstrated a promising potential application as a flexible supercapacitor in portable electronic devices. Similar works reported on the mechanical flexibility of electrode materials upon straight-bending process, but none of them measured the performance of electrode materials upon continuous GCD cyclic stability, as summarised in Additional file 1: Table S1.

## Conclusions

A flexible and bendable supercapacitor device was fabricated by using a simple and low-cost two-step electrochemical deposition of PPy and rGO on the surface of carbon bundle fibre and assembled into supercapacitor devices by using them to sandwich a solid-state electrolyte. The formation of a hydrogen bond in CBF/PPy-rGO-2, as depicted in the XPS results, transferred electrons efficiently between the rGO and PPy components, leading to an excellent electrochemical performance for the symmetrical solid-state carbon bundle fibre supercapacitor. The formation of a high porosity structure in the PPy-rGO-2 efficiently increased the ionic penetration, which remarkably enhanced the specific capacitance value of 96.16 A g^− 1^ at a current density of 1 A g^− 1^. The CBF/PPy-rGO-2 supercapacitor device has a capacity retention of 71% after 500 GCD cycles, and it showed outstanding stability when subjected to bending at various angles. Therefore, these results demonstrate the feasibility of fabricating flexible supercapacitors for portable electronic devices using the simple electrochemical deposition of active materials on a carbon bundle fibre.

## Additional files

Additional file 1: Table S1. Electrochemical performances of flexible graphene-based solid-state fibre supercapacitors. (DOCX 26 kb)
