# Supplementary material for: Electrodeposition of Polypyrrole and Reduced Graphene Oxide onto Carbon Bundle Fibre as Electrode for Supercapacitor
Source: Nanoscale Res Lett. 2017 Apr 4;12:246. doi: 10.1186/s11671-017-2010-3 (PMC5380573; doi:10.1186/s11671-017-2010-3)
Supplement: Supplementary file 1 — Electrochemical performances of flexible graphene-based solid-state fibre supercapacitors. (DOCX 26 kb) [file 11671_2017_2010_MOESM1_ESM.docx]

**Additional File 1**

**Electrodeposition of Polypyrrole and Reduced Graphene Oxide onto Carbon Bundle Fibre as Electrode for Supercapacitor**

**Hamra Assyaima Abdul Bashid ^1^, Hong Ngee Lim ^1,2*^, Sazlinda Kamaruzaman ^1^, Suraya Abdul Rashid ^3,4^, Robiah Yunus ^3^, Nay Ming Huang ^5^, Chun Yang Yin ^6^, Mohammad Mahbubur Rahman ^7^, Mohammednoor Altarawneh ^8^, Zhong Tao Jiang  ^8^, Pandikumar Alagarsamy ^9^**

^1^ Department of Chemistry, Faculty of Science, Universiti Putra Malaysia, 43400 UPM Serdang, Selangor, Malaysia

^2^ Functional Device Laboratory, Institute of Advanced Technology, Universiti Putra Malaysia, 43400 UPM Serdang, Selangor, Malaysia

^3^ Department of Chemical and Environmental Engineering, Faculty of Engineering, Universiti Putra Malaysia, 43400 UPM Serdang, Selangor, Malaysia

^4^ Materials Processing and Technology Laboratory (Nanomaterials and Nanotechnology Group), Institute of Advanced Technology, Universiti Putra Malaysia, 43400 UPM Serdang, Selangor, Malaysia

^5^ Faculty of Engineering, Xiamen University of Malaysia, Jalan Sunsuria, Bandar Sunsuria, 43900 Sepang, Selangor Darul Ehsan, Malaysia

^6^ Newcastle University Singapore, 537 Clementi Road #06-01, SIT Building @ Ngee Ann Polytechnic, Singapore, 599493

^7^ Department of Physics Jahangirnagar University Savar, Dhaka 1342, Bangladesh.

^8^ Surface Analysis and Materials Engineering Research Group, School of Engineering and Information Technology, Murdoch University, Murdoch, Western Australia 6150, Australia

^9^ Research Institute & Department of Chemistry, SRM University, Kattankulathur-603 203, Chennai, India.

* Corresponding author, Email address: [hongngee@upm.edu.m](mailto:hongngee@upm.edu.m)y (Hong Ngee Lim)

**Table S1.** Electrochemical performances of flexible graphene-based solid-state fibre supercapacitors.

| No. | Electrode material | Fabrication method | Electrochemical Performance | | | Reference |
| --- | --- | --- | --- | --- | --- | --- |
|  |  |  | Specific capacitance | Mechanical flexibility | GCD cyclic stability |  |
| 1 | Graphene fibre@3D-Graphene | The GF@3D-Graphene was prepared by directly electrochemically electrolysing GO aqueous suspension on GF. The supercapacitor was built by intertwining two electrodes with electrolyte (H_2_SO_4_-PVA). | 1.2-1.7 $\mathrm{mF}\mathrm{cm}^{-2}$. | Stable capacitance of 30-40 μF after 500 straight-bending cycles at applied current 2 μA. | Not reported | [[1](#_ENREF_1)] |
| 2 | Graphene-MnO_2_ | Formation of core-sheath graphene on graphene fibre by electrolysing GO suspension, followed by electrodeposition of MnO_2_ nanostructures. The supercapacitor was fabricated by intertwining two electrodes pre-coated with electrolyte (H_2_SO_4_-PVA). | 9.1-9.6 $\mathrm{mF}\mathrm{cm}^{-2}$. | Stable capacitance of 70-73 μF after 1000 cycles of the straight-bending-straight process at applied current 2 μA. | Not reported | [[2](#_ENREF_2)] |
| 3 | Graphene-PPy | GO-PPy was prepared by directly spun GO-pyrrole into an aqueous FeCl_3_ solution, in which pyrrole was polymerised to PPy, and then followed by chemical reduction to prepare Graphene-PPy. Two fibre electrodes were intertwined with electrolyte (H_2_SO_4_-PVA). | 107.2 $\mathrm{mF}\mathrm{cm}^{-2}$at current density of 0.24 $\mathrm{mA}\mathrm{cm}^{-2}.$ | Stable capacitance of 95-105 $\mathrm{mF}\mathrm{cm}^{-2}$in the bending or straight status during 1000 cycles at current density of 0.15 $\mathrm{mA}\mathrm{cm}^{-2}.$ | Not reported | [[3](#_ENREF_3)] |
| 4 | CBF/PPy-rGO-2 | PPy-rGO-2 nanocomposite was electrochemically deposited on CBF, which acted as an electrode. Two symmetrical electrodes were dipped in the solid-state electrolyte (PVA-CH_3_CO_2_K) and sandwiched together side by side. | 96.2 $F g^{-1}$ at current density of 1 $A g^{-1}.$ | Insignificant effect on the capacitance values bending at various angles. | 71% after 500 cycles of GCD at current density of 1 $A g^{-1}.$ | This work |

**References**

1. Y. Meng, Y. Zhao, C. Hu, H. Cheng, Y. Hu, Z. Zhang, G. Shi and L. Qu, "All-graphene core-sheath microfibers for all-solid-state, stretchable fibriform supercapacitors and wearable electronic textiles," *Advanced Materials*, vol. 25, no. 16, pp. 2326-2331, 2013.

2. Q. Chen, Y. Meng, C. Hu, Y. Zhao, H. Shao, N. Chen and L. Qu, "MnO_2_-modified hierarchical graphene fiber electrochemical supercapacitor," *Journal of Power Sources*, vol. 247, pp. 32-39, 2014.

3. X. Ding, Y. Zhao, C. Hu, Y. Hu, Z. Dong, N. Chen, Z. Zhang and L. Qu, "Spinning fabrication of graphene/polypyrrole composite fibers for all-solid-state, flexible fibriform supercapacitors," *Journal of Materials Chemistry A*, vol. 2, no. 31, pp. 12355-12360, 2014.
